# RAP1/TERF2IP—A Multifunctional Player in Cancer Development

**DOI:** 10.3390/cancers13235970

**Published:** 2021-11-27

**Authors:** Anna Deregowska, Maciej Wnuk

**Affiliations:** 1 Department of Biotechnology, Institute of Biology and Biotechnology, College of Natural Sciences, University of Rzeszow, Pigonia 1, 35-310 Rzeszow, Poland; 2 Department of Biology, Institute of Biology and Biotechnology, College of Natural Sciences, University of Rzeszow, Pigonia 1, 35-310 Rzeszow, Poland

**Keywords:** TERF2IP, telomere, DDR response, NF-κB signaling, cancer

## Abstract

**Simple Summary:**

RAP1 (TERF2IP) is a member of the shelterin complex that protects telomeric DNA and plays a critical role in maintaining chromosome stability. However, mammalian RAP1 was recently found to have additional functions apart from telomeres, acting as a regulator of the NF-κB pathway and transcription factor, and has been suggested that they have putative roles in cancer development. Here, we focus on the main roles of RAP1 in different mechanisms of oncogenesis, progression, and chemoresistance, and consider the clinical significance of findings about its regulation and biological functions.

**Abstract:**

Mammalian RAP1 (TERF2IP), the most conserved shelterin component, plays a pleiotropic role in the regulation of a variety of cellular processes, including cell metabolism, DNA damage response, and NF-κB signaling, beyond its canonical telomeric role. Moreover, it has been demonstrated to be involved in oncogenesis, progression, and chemoresistance in human cancers. Several mutations and different expression patterns of RAP1 in cancers have been reported. However, the functions and mechanisms of RAP1 in various cancers have not been extensively studied, suggesting the necessity of further investigations. In this review, we summarize the main roles of RAP1 in different mechanisms of cancer development and chemoresistance, with special emphasis on the contribution of RAP1 mutations, expression patterns, and regulation by non-coding RNA, and briefly discuss telomeric and non-telomeric functions.

## 1. Introduction

Mammalian telomeres are specialized nucleoprotein complexes, consisting of several kilobases of double-stranded DNA with the hexameric repetetive sequence 5‘-TTAGGG/3‘-AATCCC in vertebrates, the telomeric repeat-containing RNA (*TERRA*), and the specific, telomere-associated shelterin proteins. Of note, at the 3’end of linear chromosomes, a 150–300 nucleotide G-rich single-stranded 5‘-TTAGGG-3‘ sequence extends beyond the double-stranded telomeric DNA in human cells [1]. It is believed that this single-stranded extension plays a crucial role for proper telomere function, potentially by promoting the formation of telomeric T-loop/D-loop structure [1,2]. These structures maintain genome integrity by capping the chromosome terminus protecting them from end-to-end fusion, degradation, or recombination [1]. In most mammalian somatic cells, the telomere length decreases after each cell division. Telomere shortening below a critical level of telomeric sequence repetition leads to cell cycle arrest, and senescence or apoptosis [2]. Therefore, some cells, such as germ cells, stem cells, or cancer cells, have mechanisms that help maintain the appropriate telomere length. These cells may have an active enzyme with reverse transcriptase activity called telomerase [3]. As a ribonucleoprotein, telomerase promotes telomere elongation in each round of DNA replication in cancer cells. Some cancer cells also use another telomerase-independent mechanism known as alternative mechanisms of telomere lengthening (ALT). It is worth noting that in cancer cells both mechanisms of telomere elongation telomerase-dependent and telomerase-independent can also coexist, as both allows for rapid cell proliferation and immortality [2,3].

RAP1 (also known as TERF2IP) is a member of the shelterin complex that protects telomeric DNA and plays a critical role in maintaining chromosome stability. The telomeric complex is composed of five other proteins, namely TRF1, TRF2, TIN2, TPP1, and POT1, and it is important for managing the regulation of telomere length and the protection of telomeres from degradation, aberrant recombination, inappropriate processing by the DNA-repair pathway, and the facilitation of chromosome capping to mediate telomerase activity. The loss or mutation of shelterin proteins results in uncapped telomeres, which activate DNA damage response pathways and trigger the degradation or fusion events of chromosomes [4]. However, RAP1 is the most conserved of all shelterin proteins and plays pleiotropic functions not only in telomeres but also in regulating the DNA damage response, inflammation, metabolism, and oxidative stress [5,6,7,8]. Here, we discuss the function of RAP1 in light of already known data about the role of RAP1 in human cancers.

## 2. Structure of Human RAP1 Transcripts and Protein Variants

Human RAP1 is encoded by a gene named *TERF2IP* situated on chromosome 16q23.1 that consists of three exons. Human RAP1 was identified as an ortholog of *Saccharomyces cerevisiae* telomere-binding protein (ScRAP1) with a yeast two-hybrid screen of HeLa cDNA with part of the human TRF2 as a bait protein [9]. In *Saccharomyces cerevisiae*, ScRAP1 is a docking platform for other telomere-associated proteins, including Rif family proteins (Rif1 and Rif2) crucial to telomere length regulation and Sir family proteins (Sir3 and Sir4) important to gene silencing [10,11]. *Rap1* mutants in *Saccharomyces cerevisiae* are defective in telomere length control and telomere position effects [12].

Human RAP1 can be translated from five spliced transcripts and is expressed in four isoforms, which contain one well-described isoform (399 aa) with a molecular weight of approximately 47 kDa, and three potential isoforms that are computationally mapped (342 aa, 122 aa and 99 aa) (Table 1).

**Figure 1:**
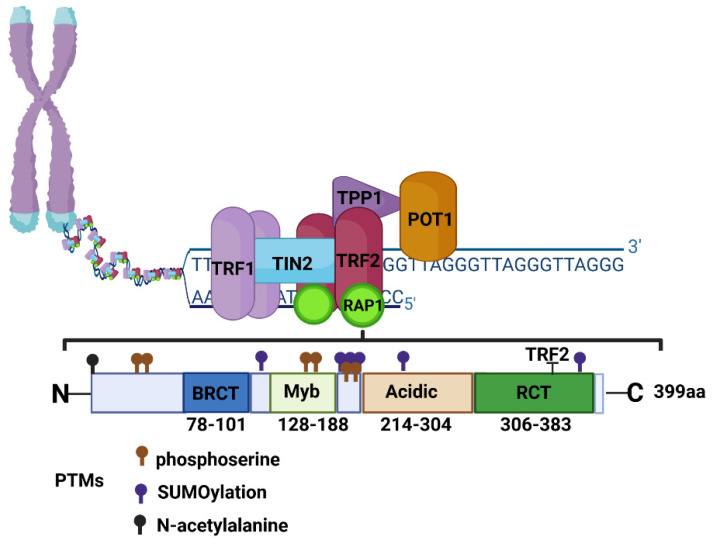
Schematic of the shelterin complex and the structure of RAP1 protein. Shelterin complex is composed of six proteins including TRF1, TRF2, TIN2, TPP1, POT1, and RAP1, which participate in telomere protection and length regulation. TRF1 and TRF2 form homodimers and bind to the dsDNA, while POT1 binds to ssDNA and forms a heterodimer with TPP1. TIN2 has the capacity to bind TRF1, TRF2, and TPP1-POT1 heterodimer and stabilizes shelterin structure, while RAP1 is recruited to telomeres through interacting with TRF2. The human RAP1 protein contains: BRCT-breast cancer susceptibility protein domain on the C-terminus, Myb motif, acidic coil−coil domain, and RCT-RAP1-specific protein-interaction domain, acidic region, and nuclear localization signal (NLS). Amino acid modifications have been marked according to UniProtKB-Q9NYB0 (TE2IP_HUMAN). Created with BioRender.com (accessed on 22 October 2021).

RAP1 contains three conserved sequence motifs: the N-terminal domain (BRCT), myeloblastosis (Myb), RAP1 C-terminal domain (RCT), and nuclear localization signal (*NLS*). Between the Myb and RCT domains lies an acidic region whose functional significance remains unclear [9,10,11] (Figure 1).

The BRCT domain, which is recognized in many DNA damage response proteins, contains two layers of helices: a bottom layer with three helices and a top layer with another two helices. The deletion of BRCT from human RAP1 has been reported to decrease telomeric length heterogeneity, indicating that RAP1 may also play a role in the regulation of human telomere length distribution [13]. Deletion of the BRCT domain from human RAP1 has been reported to reduce telomere length heterogeneity [14].

The Myb domain is a double-stranded DNA binding motif that consists of a three-helix bundle. In budding yeast ScRAP1 binds to telomeric dsDNA via its tandem Myb domains; however, mammalian RAP1 is recruited to telomeres through binding to TRF2. Human RAP1 contains one Myb domain but does not have any DNA-binding activity due to the lack of significant positively charged residues on its surface [14]. In vitro studies by Arat and Griffith (2012) suggested that human RAP1 can bind to DNA directly in a sequence-independent manner through the BRCT domain [15]. However, in vivo studies in mammalian cells have not been able to prove these results and demonstrated that RAP1 relies completely on its interaction with TRF2 [15].

The RCT domain is a three-helix bundle that recognizes a helical peptide from binding partners, such as TRF2 driven by hydrophobic interactions, which thus tethers it to the telomeres.

It is worth noting that the domains of RAP1 allow the interaction of this protein with other molecular partners than just members of the shelterin complex [5,6].

## 3. RAP1 Mutations in Human Disease

**Figure 2:**
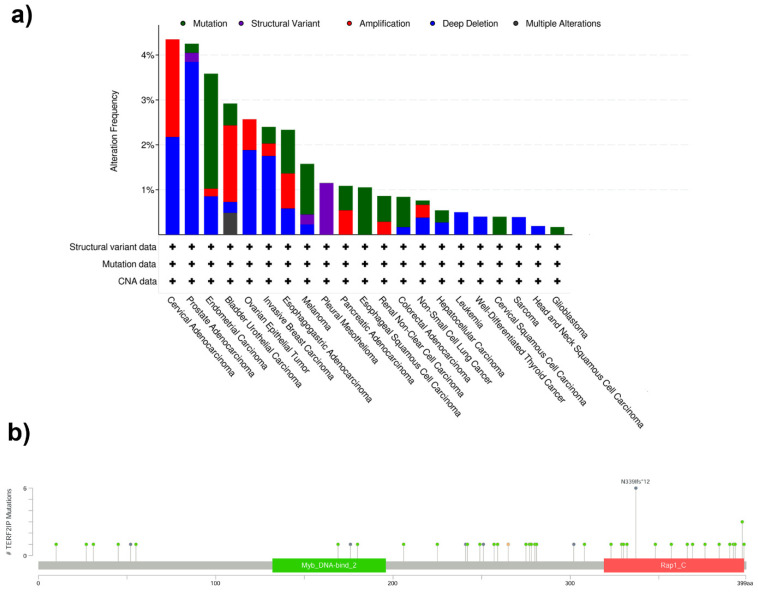
The genetic alterations identified in *TERF2IP* gene according to cBioPortal [16]. (**a**) Alteration gene frequency including single nucleotide missense mutation (green), structural variant (purple), gene amplification (red), deletion (blue) and multiple genetic alteration (gray) in the various type of cancers. (**b**) Localization of genetic mutation in domain of *TERF2IP* gene. Colors mean the following genetic alterations: single nucleotide missense mutation (green), splice (orange), truncating variants (gray). Created with cBioPortal (cbioportal.org, accessed on 21 November 2021).

To date, over 50 genetic alterations have been identified in *TERF2IP* gene (Table S1) according to cBioPortal [16]. The above genetic changes in the *TERF2IP* gene, including single nucleotide mutation, structural variation, gene amplification and/or deletion of *TERF2IP*, were noted and have been reported in various type of cancers (Figure 2a). The most frequent missense mutation was observed in endometrial carcinoma, melanoma, and esophageal squamous cell carcinoma (Figure 2a). The abovementioned genetic alterations have been detected in all domains of RAP1 (Figure 2b) [17,18,19,20,21]. It is also worth adding that assessment of a gene damage index for all protein-coding genes places *RAP1* in the top 20% of human genes concerning mutation intolerance [22]. Moreover, *RAP1* is classified among the top 10% of ‘functional indispensability’ human genes, a characteristic that incorporates gene centrality, structural information, and evolutionary constraints [23].

## 4. Dysregulation of RAP1 Expression in Cancer

RAP1 is a telomeric protein that is expressed in all analyzed human tissues by low tissue specificity and plays a role in both the nucleus and cytoplasm [24]. Moreover, recent studies suggest the functional involvement of telomeric genes, including *RAP1* in promoting cancer and *RAP1* expression, would be beneficial for the selection of a unique and reliable cancer biomarker. Several groups have shown that *RAP1* is highly variable across cancer types, and also in some cases within cancer types. Accordingly, the overexpression of *RAP1* has been reported in breast cancer [25], gastric carcinoma [26], non-small cell lung cancer [27], mantle cell lymphoma [28], multiple melanoma [29], colorectal cancer [30], and renal cell carcinoma [31]. In familial papillary thyroid cancer [32], reduced expression of *RAP1* has been described. For chronic lymphocytic leukemia (CLL), the literature presents conflicting results [33,34]. Furthermore, it is postulated that RAP1 levels are predictive of the success of chemotherapy in breast and colon cancer [30,35].

Bhari et al. (2021) reported the prognostic value of telomeric complex genes (TRF1, TRF2, RAP1, POT1, TIN2, and TPP1) in human breast tumors. They found that mRNA expression levels of RAP1 were markedly increased in breast tumors compared to adjacent non-malignant tissues. However, they are not associated RAP1 levels with relapse-free survival (RFS) in breast cancer patients [25]. Other group of researchers showed that higher RAP1 levels are correlated with poor prognosis for patients undergoing chemotherapy in breast cancer patients. Interestingly, they found increased levels of the nuclear fraction of the RAP1 protein after treatment with camptothecin [35]. The abovementioned results suggest that RAP1 could be predictive of chemotherapy in breast cancer outcomes.

In a study of pattern expression of *MRE11*, *RAD50*, *NBS1*, and *RAP1* in gastric carcinomas, Matsutani et al. (2001) found increased expression of *RAP1* in gastric carcinomas compared to their non-neoplastic counterparts in 60% of cases. Moreover, gastric carcinomas with high *TRF2* expression expressed significantly higher levels of *RAP1* than those with low *TRF2* expression [26]. In Panero et al.’s study of the expression profile of telomere-related genes (*TRF1*, *TRF2*, *POT1*, *TPP1*, *TIN2*, *RAP1*, and *DKC1*) in mantle cell lymphoma, significantly increased expression of all analyzed genes compared with healthy individuals was observed [28]. In addition, Panero’s group studied the expression profile of shelterin genes (*POT1*, *TIN2*, *RAP1*, and *TPP1*) in plasma cell disorders. It was observed that multiple melanoma patients expressed higher levels of shelterin genes with respect to monoclonal gammopathy of undetermined significance [29]. Xiao et al. (2017) analyzed both the expression and the localization of RAP1 in high-grade non-small cell lung cancer (NSCLC) and found an overall higher mRNA and protein expression of RAP1 in NSCLC cells, particularly in the cytoplasmic fraction, compared with normal lung epithelial cells. Moreover, a higher level of cytoplasmic *RAP1* was associated with a higher grade of NSCLC, suggesting that RAP1 may be an indicator of high-grade NSCLC and has a critical role in lung cancer progression [27]. According to Anuja et al. (2020), higher *RAP1* expression was observed in the tumors of colorectal patients. Dataset analysis revealed that the high expression of RAP1 indicates its possible role in promoting radio resistance in colorectal cancer and that it is associated with a poor prognosis [30]. A significant increase in the expression of RAP1 was also noted in renal cell carcinoma (RCC) tissue compared to normal renal parenchyma [31].

As reported in familial papillary thyroid cancer, a significant reduction in mRNA expression of *POT1* and *RAP1* compared with patients with sporadic papillary thyroid cancer was found [32]. In the pathogenesis of B cell-chronic lymphocytic leukemia, Poncet et al. (2008) described global deregulation of telomere-associated genes, with 3-fold reduced mRNA levels of *RAP1* compared to healthy donors [33]. In contrast, Hoxha et al. (2014), using microarray analysis, observed *RAP1*, *TRF1*, and *POT1* overexpression in B-cell-chronic lymphocytic leukemia. Apparently, conflicting results could arise due to the number and clinical characteristics of patients [34].

Recently, our group determined whether the BCR/ABL1 copy number, expression, and/or activity were responsible for telomere maintenance and the deregulated expression of selected shelterin components (TRF1, TRF2, RAP1, and POT1) in widely used chronic myeloid leukemia (CML) cell lines. We found that the expression of RAP1 and POT1 at the protein level was positively correlated with the levels of expression and activity of *BCR/ABL1,* suggesting that *BCR/ABL1* kinase expression and activity may upregulate the levels of RAP1 and POT1 and play a crucial role in the maintenance of telomeres in CML cells [36]. The differential expression of RAP1 in selected human cancers is summarized in Table 2.

## 5. Role of Non-Coding RNA in the Regulation of RAP1

Non-coding RNAs (ncRNAs) are a large segment of the transcriptome that do not have apparent protein-coding roles but were found to participate in the regulation of multiple biological processes, including cancers [37,38,39]. Based on their length, ncRNAs can be divided into two categories: ncRNAs shorter than 200 bp, such as microRNAs (miRNAs), piwi-interacting RNAs (piRNAs), small nucleolar RNAs (snoRNAs), small nuclear ribonucleic acid RNAs (snRNAs), transfer RNAs (tRNAs), and ncRNAs longer than 200 bp called long non-coding RNAs (lncRNAs) [40]. miRNAs are a class of small non-coding RNAs (20–24 nucleotides) involved in the regulation of target gene expression at the post-transcriptional level by binding to the 3′-untranslated region (3′UTR) of downstream target genes and inhibiting their use through either degradation or translational repression. miRNAs control the expression of approximately 30% of genes involved in a wide array of biological processes, and they have been found to be heavily dysregulated in all cancer types studied [41,42,43]. Qian et al. (2020) demonstrated that a miR-1246-dependent mechanism induced M2 macrophage polarization by directly targeting RAP1 in glioma patients. Functional analyses indicated that the overexpression of miR-1246 significantly inhibited RAP1 expression at both the mRNA and protein levels, driving NF-κB pathway inhibition and STAT3 pathway activation, which promoted the proliferation and metastasis of gliomas. Target specificity was found between miR-1246 and the RAP1 3′UTR, as evidenced by a luciferase reporter assay. Moreover, they determined that RAP1 expression was negatively associated with glioma patient survival [44]. The regulation of RAP1 by miRNAs was previously reported by Chilton et al. (2014), who analyzed the exercise-induced expression patterns of miRNAs and changes in genes involved in telomere regulation in white blood cells. RAP1 was identified as a potential binding target for miR-186 and miR-96 and was demonstrated to accompany downregulation after intense cardiorespiratory exercise, suggesting that exercise of appropriate intensity may mediate improved telomere homeostasis [45]. Regarding cancer, miRNAs provide a new powerful avenue for the discovery of novel genetic risk factors. However, the association between the expression levels of various miRNAs and RAP1 in cancers is not yet clearly defined. A list of miRNAs involved in RAP1 regulation predicted by miRDB (http://mirdb.org, accessed on 15 September 2021) is presented in Table 3.

Like miRNAs, lncRNAs have been functionally associated with human diseases in particular cancers. The majority of long non-coding transcripts ranging in length from 200 bp to ~100 kilobases are transcribed by RNA polymerase II, and most of them are also polyadenylated at the 3′ end. LncRNAs are functionally very diverse, and they are therefore involved in the regulation of gene and genome activity via multiple mechanisms, including histone modifications, DNA methylation, chromatin remodeling, and regulation at transcriptional, post-transcriptional, and translational levels [46,47].

Dysregulated lncRNAs participate in the occurrence and development of diseases, especially in tumors. It is postulated that cancer-type-specific expression of lncRNAs may be useful as the diagnostic or survival prediction markers [48,49,50]. Although an increasing number of lncRNAs have been characterized and are shown to be involved in the development of many cancers, the functions of the great majority of lncRNAs and their downstream targets remain unknown. Therefore, bioinformatics tools and databases able to take advantage of the increasing amount of publicly available data to predict RNA–RNA interactions and cross-talk between lncRNAs could be very useful [51].

The LncRNA2Target v.3.0 database (http://bio-annotation.cn/lncrna2target/, accessed on 15 September 2021) was employed to identify RAP1 as a target gene regulated by lncRNAs. The differentially expressed RAP1 was inferred from the lncRNA knockdown or overexpression experiment followed by microarray or RNA-seq and is summarized in Table 4.

## 6. The Role of RAP1 in Telomere Length Regulation

Telomere integrity is maintained through the involvement of the shelterin and cellular multiprotein complex (CST) [4,61]. However, research over the past decade has shown that the contribution of shelterin proteins to the regulation of telomere length is more complex than previously thought.

**Figure 3:**
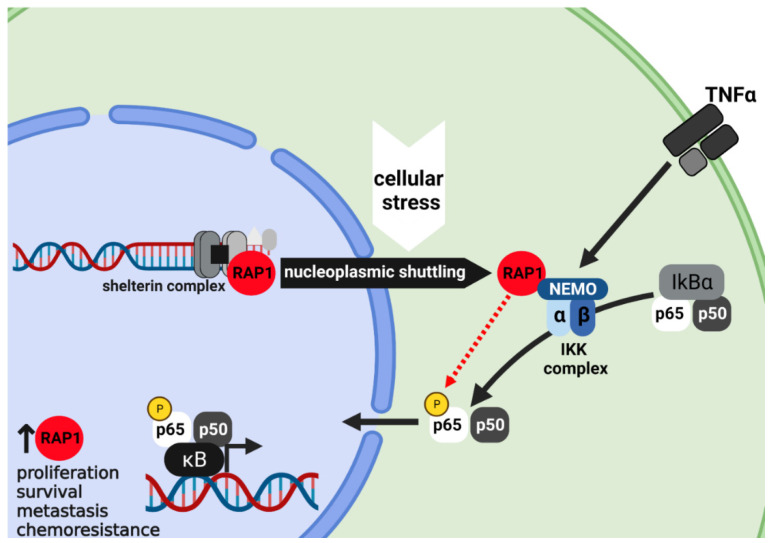
Schematic representation showing the role of RAP1-enhanced activation of NF-κB signaling. In cancer cells under extreme conditions, such as oxidative stress, RAP1 shuttles from nucleus to cytoplasm, where RAP1 forms a complex with IKK complex. RAP1–IKK interaction is essential for phosphorylation of the p65 subunit at Ser 536 (red dotted arrow) to make it transcriptionally competent. RAP1-mediated activation of NF-κB can stimulate transcription of genes involved in inflammation, proliferation, tumor survival, metastasis, and chemoresistance. Moreover, activation of NF-κB is correlated to an increase in RAP1 levels. Created with BioRender.com (accessed on 6 October 2021).

Recent studies have shown that the role of mammalian RAP1 in telomere length regulation is more unclear than, e.g., in budding yeast cells [62,63]. Under normal conditions, mammalian RAP1 associates with the TRF2 protein and enhances the selectivity of TRF2 to telomeric DNA and TRF2 localization to single–double-strand DNA junctions. Janoušková et al. (2015) found that RAP1 neutralizes the electrostatic attraction of TRF2 to DNA and improves its binding selectivity toward telomeric DNA [64]. RAP1, as a partner of TRF2 in the telomere, is also removed upon the deletion of TRF2 [65]. RAP1 abrogation does not disrupt the binding of other shelterins to telomeres [66]. Moreover, deletion of RAP1 in the telomere does not affect cell viability or lead to telomere dysfunction in vivo. In support of this notion, *Rap1* knockout mice are alive and fertile and show no change in telomere length, even after three generations [22]. Indeed, it has recently been shown that mice lacking both RAP1 and telomerase show telomere shortening and progressively decreased survival with increasing mouse generations in comparison to telomerase single mutants, demonstrating an unanticipated genetic interaction between telomerase and RAP1 [67]. Furthermore, Robinson et al. (2021) uncovered a mechanism based on an RAP1 SUMOylation that coordinates telomere maintenance through a recombination-based telomere maintenance mechanism called alternative lengthening of telomeres (ALT). The authors found that in breast cancer and osteosarcoma cell lines that maintain their telomere length through ALT, the telomere-associated protein SLX4IP promotes RAP1 SUMOylation by PIAS1, driving the detachment of RAP1 from telomeres and shuttling from the nucleus to the cytoplasm, which enhances its extratelomeric function. In the cytoplasm, RAP1 is involved in the activation of the transcription factor NF-κB by binding to IκB kinase (IKK) and the induction of Jagged-1 expression, which promotes Notch signaling pathways that favor ALT [68] (Figure 3).

The removal of RAP1 from several human cancer cell lines, including cervical cancer (HeLa), fibrosarcoma (HT1080), and colon cancer (HTC116), through transcription activator-like effector nuclease (TALEN) did not appear to affect telomere length dynamics [22]. Moreover, the deletion of RAP1 also did not induce an obvious change in telomere length heterogeneity. Interestingly, O’Connor et al. (2004) showed that the knockdown of *RAP1* expression by small hairpin interference RNA displayed a telomere elongation phenotype [69]. It is unclear why TALEN-mediated RAP1 knockout or partial RAP1 inactivation by shRNA showed contrary findings, and the role of RAP1 in telomere length control still requires further investigation.

Interestingly, Braig et al. (2014) reported that telomere shortening in the progression of CML is accompanied by a telomere-associated secretory phenotype (TASP), and the authors suggested that RAP1 might activate candidate NF-κB-dependent genes (e.g., *CCL4*, *IL-6*, *IL-8*) responsible for the inflammatory environment in cells with short, but not critically short telomeres [70]. In addition to its role in regulating telomere length, RAP1 deficiency has also been reported to induce the expression of *TERRA*, which can inhibit telomerase activity by binding to both telomerase subunits, including reverse transcriptase (TERT) and telomerase RNA moiety (TERC) [71], or interact with the EXO1-inhibiting Ku70/80 heterodimer, which promotes EXO1-dependent telomere shortening [72]. In accordance with these findings, Zhang et al. (2019) demonstrated that RAP1 negatively regulated *TERRA* expression in human mesenchymal stem cells (hMSCs), suggesting that RAP1 counteracted telomere length in hMSCs [73]. Guanine-rich *TERRA* can form telomeric RNA–DNA hybrids known as a telomeric R-loops and may actively engage in homologous recombination in telomerase-negative yeast and human ALT-positive cancer cells [74,75]. Targeting TERRA in tumor cells could be an attractive strategy for cancer therapy due to its role in inhibiting telomerase activity and ALT-mediated telomere elongation; however, it needs further investigation.

## 7. Telomeric and Non-Telomeric Roles of RAP1 in the DNA Damage Response

In healthy cells, chromosome ends resemble double-stranded DNA breaks, but they do not activate a damage response. It is well known that disruption of shelterin components leads to activation of the DNA damage response (DDR), including ATR- and ATM-dependent signaling pathways, which involve homology-directed repair (HDR) and non-homologous end-joining (NHEJ) mechanisms [76,77]. HDR results in telomere sister chromatid exchanges (T-SCE), the formation of telomere-free chromosome ends, and loss of telomeric DNA, while NHEJ results in chromosome end-to-end fusions [78,79,80].

RAP1 binds telomeric DNA through TRF2, which has been found to have an essential role in the formation of the T-loop, a secondary structure that hides chromosome ends, thereby preventing ATM activation and NHEJ by blocking the loading of the Ku70/Ku80 heterodimer at telomeres [79,81,82,83]. Electron microscopy analysis of T-loop formation revealed that the TRF2-RAP1 complex possesses a greater ability to remodel telomeric DNA than either component alone [15]. Although budding yeast scRAP1 is a key chromosome-end anti-fusion factor [63], inconsistent findings concerning its role in telomere protection in mammalian cells have been reported.

Mouse and human telomeres lacking RAP1 do not disrupt the binding of other shelterins to telomeres or develop DNA damage response activation but are prone to recombination by HDR, suggesting that TRF2 alone is sufficient to repress the NHEJ pathway and ATM kinase activity at telomeres. In contrast with its role in the ATM and NHEJ pathways, RAP1 is critical to repress aberrant HDR at telomeres which can alter telomere length. It has been reported that RAP1 is lacking in the absence of Ku70/80 in mouse embryonic fibroblasts (MEFs) in HDR activation at telomeres, resulting in telomere recombination and T-SCE events without the induction of telomere dysfunction-induced foci (TIFs) [84]. Additionally, Rai et al. (2016) showed that RAP1-TRF2 is required to fully repress poly(ADP-ribose) polymerase 1 (PARP1) and structure-specific endonuclease subunit (SLX4) localization at telomeres and further T-loop resolution and telomere loss due to circle-mediated excision, thus repressing HDR. Molecularly, in MEFs with RAP1-TRF2 heterodimer deletion, telomeric accumulation of PARP1 and SLX4 was observed. This is accompanied by rapid telomere resection and the generation of telomere-free chromosomal fusions. Thus, these findings demonstrated the biological importance of RAP1 in telomere end protection by repressing the aberrant HDR pathway [65].

Interestingly, Martinez et al. (2016) observed that the lack of functional Rap1 enhanced telomere fragility and recombination in a telomerase RNA component (TERC)-deficient mice with short telomeres. However, they pointed out that RAP1 activity in telomere maintenance depends on telomere length, and it is especially essential in cells with telomerase deficiency [67]. A protective role of RAP1 against the effects of short telomeres was also documented by Lototska et al. (2020) They showed that the lack of functional RAP1 in human cancer cells leads to telomere fusions only in cells without active telomerase. Using human primary lung fibroblasts, they also proposed a mechanism of preventing telomere fusion by RAP1 that depends on inhibiting p53-binding protein 1 (53BP1) binding to telomeres [85].

Moreover, emerging evidence has suggested that due to several interaction domains, mammalian RAP1 may also play non-telomeric function as an adaptor in the NHEJ pathway, apart from the telomere [35]. O’Connor et al. (2004) demonstrated that RAP1, independent of TRF2, is able to interact with DDR proteins such as Rad50/Mre-11 and Ku86 [69]. Another group found that the efficiency of NHEJ was correlated with RAP1 activity in a breast cancer cell model (MCF-7) [35]. They showed that RAP1 plays a role as an adaptor in promoting the association of XRCC4/Ligase IV with DNA-PK. Moreover, *Rap1* knockout in transgenic mice with c-Myc overexpression promoted tumorigenesis. Interestingly, in the absence of Rap1, accelerated lymphomagenesis and increased tumor sizes were observed. Another interesting observation was that reduction in Rap1 levels, as well as loss of Rap1, sensitized the mouse to double-strand breaks caused by irradiation and 5-fluorouracil (5-FU) [35].

## 8. Non-Canonical Role of RAP1 in Human Cancers

Mammalian RAP1 was recently found to have additional functions apart from telomeres by acting as a regulator of the NF-κB pathway and transcription factor, and it has been suggested to have putative roles in cancer development [6,66].

The first such evidence was provided by Teo et al. (2010), who demonstrated that RAP1 can translocate to the cytoplasm and that, independently of TRF2, it acts as a modulator of NF-κB signaling pathway, thus affecting NF-kB activity, which also has an important role in cancer. It has been reported that the RAP1–NF-κB axis promotes the invasion of breast cancer cells. Mechanistically, they described that RAP1 could function as an adaptor that specifically directs and regulates the kinase activity of IKK complexes via phosphorylation of the p65 subunit at Ser536 and therefore enhances NF-kB signaling [6]. Additionally, Swanson et al. (2016) found that RAP1 levels in the nucleus decreased, but those in the cytoplasm remained stable when the cells were treated with H_2_O_2_ or serum-depleted medium, suggesting the possible role of RAP1 in the response to oxidative stress in the aging of human dermal fibroblasts derived from neonatal foreskin. Similar results were obtained using immortalized glioblastoma cells (U251), thus indicating that under such extreme conditions, nuclear RAP1 translocates to the cytoplasm to maintain the cytoplasmic pool for continued NF-κB signaling in aging cells as well as cancer cells [86]. Constitutive NF-κB activation has also been associated with resistance to chemotherapy [87]. Interestingly, similar to inhibiting NF-κB [88], RAP1 inactivation by siRNAs sensitizes breast cancer cell lines to apoptosis induced by TNFα (MCF7, BT474, MDA-MB-231) and adriamycin (BT474, MDA-MB-231), and this correlated with the loss of p65 phosphorylation at Ser536 in these cells. Moreover, RAP1-deficient mice showed defective NF-κB activation and displayed resistance to lipopolysaccharide-induced endotoxic shock [6]. In agreement with this finding, Xiao et al. (2017) reported that RAP1, which is highly expressed in NSCLC cells, facilitates NF-κB activation and subsequently induces BCL-2 expression, thus mediating the resistance of NSCLC cells to cisplatin [27]. Taken together, these data confirm that RAP1 could be considered a candidate gene for further research on cell sensitivity to chemotherapy treatment (Figure 3).

The second non-telomeric function of RAP1 was reported by Martinez et al. (2010), who demonstrated that RAP1 binds to both telomeric and extratelomeric TTAGGG repeats, which could be associated with subtelomeric gene promoters as well as promoters of genes involved in cell metabolism, adhesion, and tumorigenesis, indicating an important role of RAP1 in transcriptional gene regulation in mammals. They observed that RAP1-deficient MEFs exhibited the upregulation of cancer-related proteins, such as *NNMT*, *H19*, *IFI27*, *SLIT3*, *CTGF*, and *GRIA3*, while they exhibited the downregulation of *STMN2* [66]. In accordance with the role of RAP1 in cell metabolism, Ferrara-Romeo et al. (2018) showed that RAP1-deficient mice have increased hepatocellular carcinoma susceptibility, which is more acute in females, and upon treatment with carcinogen (diethylnitrosamine), they developed malignant tumors associated with reduced survival in comparison to wild-type mice [89]. Thus, a growing body of evidence strongly suggests RAP1 as a promising target for cellular metabolic networks studies. However, signaling mechanisms within RAP1-mediated metabolism-related functions remain unexplained.

## 9. Conclusions

**Figure 4:**
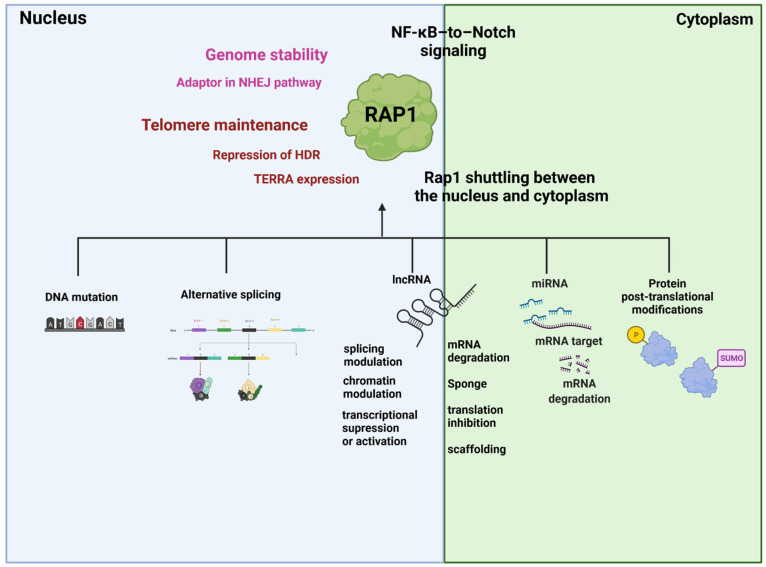
RAP1 and its regulation and role in cancer cell. RAP1 can be regulated at transcription level by alternative splicing or post-transcriptional level by miRNAs, lncRNAs or post-translational, such as enzymatic or non-enzymatic modifications. At telomeric regions, RAP1 associates with TRF2 protein, whereas TRF2–RAP1 complex represses homology-directed repair (HDR) by inhibiting PARP1 and SLX4. RAP1 regulates the expression of TERRA and in consequence influences telomere length. At non-telomeric regions, RAP1 acts as an adaptor promoting association of XRCC4/Ligase IV with DNA-PK, thus activating the non-homologous end-joining pathway (NHEJ). RAP1 acts as a transcription factor and regulates gene expression involved in cell metabolism, inflammation, and cancer. Under extreme conditions, RAP1 shuttles from nucleus to cytoplasm, where it acts as an essential modulator of NF-kB Notch-mediated pathways. Created with BioRender.com (accessed on 15 September 2021).

During the past decade, substantial evidence has accumulated showing that, apart from its telomere-associated functions, RAP1 may have other biologically important functions including regulating genes involved in metabolism, cell adhesion, the DNA damage response, and cancer progression. Given the evidence that RAP1 exerts its tumor-promoting roles via the transcriptional modulation of the NF-κB-Notch signaling cascade, therapeutic approaches that aim to impair the circuits between RAP1 and NF-κB-Notch can be devised as a strategy to check tumor growth and survival. Most recent studies have focused on RAP1 regulation at the transcriptional level. Post-transcriptional and -translational regulation is particularly important and interesting. NcRNAs, such as miRNAs or lncRNas, and post-translational regulation by enzymatic or non-enzymatic modifications, have not been extensively studied and are still being uncovered (Figure 4). However, the molecular mechanisms underlying RAP1 regulation and the functional significance of genetic mutations in cancer progression and drug resistance merit further investigation to identify more efficient therapeutic strategies for cancer.

## Figures and Tables

**Table 1:** RAP1 splicing variants (Ensembl GRCh37 release 104 - May 2021 © EMBL-EBI).

Name	Transcript ID	Length [bp]	Protein [aa]	Biotype
TERF2IP-201	ENST00000300086.5	2131	399	Protein coding
TERF2IP-202	ENST00000564671.2	1266	122	Protein coding
TERF2IP-204	ENST00000653858.1	1976	342	Protein coding
TERF2IP-205	ENST00000659145.1	1140	No protein	Processed transcript
TERF2IP-203	ENST00000569234.1	639	99	Nonsense-mediated decay

**Table 2:** The differential expression of RAP1 in selected human cancers.

Disease	Reported Level	No. of Cases	Method of Study	Ref.
breast cancer	↑	3951 breast cancer patients	mRNA expression using the University of California, Santa Cruz (UCSC) XENA platform	[25]
gastric carcinoma	↑	20 primary gastric carcinomas	mRNA expression using RT-qPCR	[26]
non-small cell lung cancer (NSCLC)	↑	93 lung adenocarcinoma and 75 lung squamous cell carcinoma tissues	cytoplasmic and nuclear expression using RT-qPCR;protein expression using Western Blot	[27]
mantle cell lymphoma	↑	24 patients	mRNA expression using RT-qPCR	[28]
multiple melanoma	↑	84 patients	mRNA expression using RT-qPCR	[29]
colorectal cancer	↑	22 colorectal cancer tissues	mRNA expression using RT-qPCR; protein expression using Western Blot	[30]
renal cell carcinoma (RCC)	↑	65 patients	mRNA expression using RT-qPCR	[31]
familial papillary thyroid cancer	↓	66 patients	mRNA expression using RT-qPCR	[32]
chronic lymphocytic leukemia (CLL)	↓	42 B-CLL patients	mRNA expression using RT-qPCR	[33]
	↑	77 early stage CLL patients	mRNA expression using RT-qPCR	[34]

**Table 3:** RAP1 as a potential target for miRNAs accessed at miRDB (http://mirdb.org, accessed on 15 September 2021).

Target Rank	Target Score	miRNA Name
1	98	hsa-miR-1305
2	91	hsa-miR-627-3p
3	88	hsa-miR-6801-5p
4	88	hsa-miR-10393-3p
5	80	hsa-miR-7856-5p
6	79	hsa-miR-570-3p
7	77	hsa-miR-6829-3p
8	77	hsa-miR-6791-3p
9	77	hsa-miR-4727-5p
10	76	hsa-miR-3065-5p
11	75	hsa-miR-585-5p
12	73	hsa-miR-3529-3p
13	73	hsa-miR-5587-5p
14	72	hsa-miR-517-5p
15	72	hsa-miR-5194
16	71	hsa-miR-6857-3p
17	67	hsa-miR-196a-1-3p
18	66	hsa-miR-637
19	66	hsa-miR-7159-5p
20	66	hsa-miR-5003-5p
21	66	hsa-miR-3120-3p
22	64	hsa-miR-6738-5p
23	64	hsa-miR-625-5p
24	64	hsa-miR-1914-3p
25	63	hsa-miR-625-3p
26	62	hsa-miR-651-3p
27	61	hsa-miR-548p
28	60	hsa-miR-3133
29	60	hsa-miR-6882-3p
30	58	hsa-miR-6857-5p
31	58	hsa-miR-10523-5p
32	57	hsa-miR-187-5p
33	57	hsa-miR-1275
34	57	hsa-miR-4477b
35	55	hsa-miR-4639-3p
36	54	hsa-miR-3922-5p
37	50	hsa-miR-450a-2-3p
38	50	hsa-miR-95-5p

**Table 4:** lncRNA2Target v.3.0 results for differentially expressed RAP1.

LncRNA Symbol	LncRNA Experiment	Reference or GEO Accession
lincFOXF1	Knockdown	[52]
NBAT1	Overexpression	[53]
LOC440173	Knockdown	[54]
DANCR	Knockdown	GSE76176
TINCR	Knockdown	[55]
HOXC-AS3	Knockdown	[56]
AF339830	Knockdown	[57]
COSMOC	Knockdown	GSE122434
lnc-Nr2f1	Overexpression	GSE125267
AK096729	Knockdown	[58]
LOC646329	Knockdown	[59]
DDGC	Knockdown	GSE158555
AC007128.1	Knockdown	[60]

## Supplementary Materials

The following are available online at https://www.mdpi.com/article/10.3390/cancers13235970/s1, Table S1: The full list of identified mutations in the RAP1 gene generated with cBioPortal [16]. In the table the following items are marked: Study of Origin, Sample ID, Cancer Type Detailed, Protein Change, Mutation Type, Variant Type, Copy variation, Chromosome localization, Start Mutation Position, End Mutation Position, Reference nucleotide, Variant nucleotide, HGVSg, HGVSc, Allele Freq (T), Allele Freq (N), Variant Reads, Reference Reads, Reference Reads (Normal), and Mutation in Sample.
